# Antibiotic treatment leads to the elimination of *Wolbachia *endosymbionts and sterility in the diplodiploid collembolan *Folsomia candida*

**DOI:** 10.1186/1741-7007-7-54

**Published:** 2009-08-24

**Authors:** Nathan Pike, Rachel Kingcombe

**Affiliations:** 1 Department of Zoology, South Parks Road, University of Oxford, Oxford, OX1 3PS, UK

## Abstract

**Background:**

*Wolbachia *is an extremely widespread bacterial endosymbiont of arthropods and nematodes that causes a variety of reproductive peculiarities. Parthenogenesis is one such peculiarity but it has been hypothesised that this phenomenon may be functionally restricted to organisms that employ haplodiploid sex determination. Using two antibiotics, tetracycline and rifampicin, we attempted to eliminate *Wolbachia *from the diplodiploid host *Folsomia candida*, a species of springtail which is a widely used study organism.

**Results:**

Molecular assays confirmed that elimination of *Wolbachia *was successfully achieved through continuous exposure of populations (over two generations and several weeks) to rifampicin administered as 2.7% dry weight of their yeast food source. The consequence of this elimination was total sterility of all individuals, despite the continuation of normal egg production.

**Conclusion:**

Microbial endosymbionts play an obligatory role in the reproduction of their diplodiploid host, most likely one in which the parthenogenetic process is facilitated by *Wolbachia*. A hitherto unknown level of host-parasite interdependence is thus recorded.

## Background

The taxonomic assemblage of endosymbiotic *Wolbachia *α-protobacteria occurs very commonly in insects, mites, crustaceans and nematodes [1,2] and is responsible for a huge variety of reproductive peculiarities. One of the most common of these peculiarities is the incompatibility of crosses between infected males and uninfected females [3], but diverse phenomena including phenotypic male feminisation [4], male killing [5], and thelytokous parthenogenesis [6] have also been conclusively attributed to *Wolbachia *infections. *Folsomia candida *(Collembola: Isotomidae) is a cosmopolitan species of springtail, which is widely used as a standard organism in toxicological assays and as a biological marker of soil pollution [7,8]. Although there have been hints that males of the species may exist [9,10], it is generally accepted that the species is made up entirely of females [8,11]. Certainly, it is well established that *F. candida *reproduces by thelytokous parthenogenesis for the vast majority of the time. From karyotype studies, we know that *F. candida *uses a diplodiploid (XX/XO) mechanism of sex determination [11,12]. *F. candida *is also host to a strain of *Wolbachia *which is unique from the strains known from other taxa [13]. The effects upon the hosts of this unique supergroup E of *Wolbachia *are yet to be characterised [14].

It has been argued that the *Wolbachia *that induces parthenogenesis may be functionally restricted to organisms which have a haplodiploid mechanism of sex determination [15]. The rationale for this argument is that, in haplodiploids, diploidisation of the unfertilised egg is all that is required to achieve sustainable parthenogenesis. Diplodiploid organisms, on the other hand, must also overcome the additional obstacle of inducing eggs to develop (a function usually performed by the fertilising sperm). Whereas we now have many demonstrations of *Wolbachia*-induced parthenogenesis in haplodiploids (for example, see Stouthamer [15] and Weeks and Breeuwer [16]), to date, we have not been able to obtain good evidence of an equivalent phenomenon in diplodiploid hosts.

We administered the antibiotics tetracycline and rifampicin *ad libitum *in the yeast food source of *F. candida *(at dry weight doses of 0% (control), 0.03%, 0.3%, 0.9% and 2.7%) over two generations. Elimination of *Wolbachia *from this host has failed in the past [13,17] but, assuming success, three outcomes were judged possible.

(1) If the *Wolbachia *are the obligatory agents of parthenogenesis or reproduction, their elimination would render all-female *F. candida *entirely sterile. The potential for *Wolbachia *to serve such a critical survival function in the biology of a diplodiploid organism has never been shown.

(2) If *F. candida *possesses an independent mechanism of parthenogenesis and is able to regulate independently reproductive developmental processes, production of females by females could continue even in the absence of *Wolbachia *(as happens in other organisms, for example, see Matsuura *et al*. [18]).

(3) If males of *F. candida *do occur (albeit very rarely), elimination of *Wolbachia *could conceivably result in production of males via a post-zygotic mechanism of sex-chromosome elimination known from another (distantly related) collembolan [19]. Of course, the common haplodiploid phenomenon of production of haploid males in the absence of *Wolbachia*-enforced diploidisation of the nucleus can be ruled out for this diplodiploid species. The discovery of males would raise the possibility of sexual reproduction, with all its genetic consequences, in a species which has hitherto been considered entirely asexual.

## Results

### *Wolbachia* infection

We randomly selected 10 adult springtails from second-generation populations of: (i) the *F. candida *control treatment (0% antibiotic); (ii) the *F. candida *high-dose (2.7%) tetracycline treatment; (iii) the *F. candida *high-dose (2.7%) rifampicin treatment; and (iv) the sibling species *Folsomia fimetaria*, which has males, reproduces sexually, and is reputed not to host *Wolbachia*. We used *Wolbachia*-specific primers in polymerase chain reaction (PCR) analyses of the total DNA extracted from each of these 40 individuals to assess whether the bacteria had been eliminated (see Methods for details of experimental procedures). *Wolbachia*-specific amplification products were not found in any springtails from the high-dose rifampicin treatment, indicating that this treatment successfully eliminated the bacteria. Similarly, none of the 10 *F. fimetaria *individuals tested positive for *Wolbachia*, and it is thus indeed likely that the two are not symbiotic. Perhaps surprisingly, all *F. candida *individuals from the high-dose tetracycline treatment tested positive for *Wolbachia*, along with all those from the control populations. The previously reported inefficacy of tetracycline in curing *Folsomia *of *Wolbachia *[13,17] is thus supported.

### Population parameters

**Figure 1:**
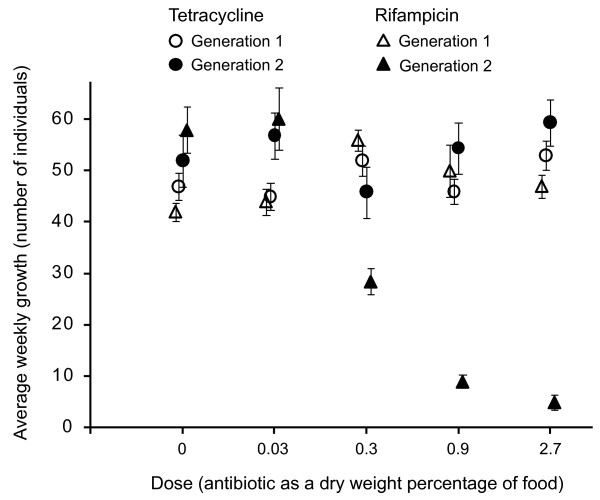
**Population growth rate (average ± standard error) measured across four doses of tetracycline and rifampicin over two generations**. Note that the *x*-axis is nominal.

Clear differences in the population growth rates of high-dose rifampicin treatments compared with high-dose tetracycline treatments were also evident, although these differences did not emerge until the second generation (Figure 1). The growth rate of rifampicin populations differed significantly between the two generations (*F*_(1,110) _= 53.5, *P *< 0.001), because of a second-generation dose effect whereby growth in the 2.7% and 0.9% treatments was significantly lower than that in the 0.3% treatments which, in turn, was lower than those in the 0.03% or 0% treatments (Figure 1). No other differences in growth rate among the treatments and/or generations were detectable.

Slight differences in growth rate, clutch size and hatching rate were found among some clones. However, as these differences did not significantly interact with the effects of interest (that is, dose, antibiotic type and generational effects), data have been pooled and clone-specific differences are not presented.

### Clutch size

At week 4 of the first-generation, individuals from the tetracycline treatments had a significantly larger mean clutch size of 44.25 ± 2.39 than individuals from the rifampicin treatments, which had a mean clutch size of 33.95 ± 1.56 (*F*_(1,118) _= 10.29, *P *= 0.002). No dose effect on clutch size was detectable in the tetracycline treatments (*F*_4,55 _= 1.79, *P *= 0.15). However, clutch size was significantly affected by rifampicin dose (*F*_4,55 _= 7.64, *P *< 0.001), reaching sizes as low as 27.00 ± 2.15 at the highest 2.7% dose.

Over the 6-month observation period (3 months in which the diet included 2.7% rifampicin followed by 3 months in which the diet was antibiotic-free), the clutch size of *F. candida *differed significantly by month (*F*_5,24 _= 5.37, *P *= 0.002). While clutch size did not differ among the first 4 months (being 27.45 ± 1.56 on average), the sizes observed in the final 2 months (40.4 ± 3.37 and 42.8 ± 2.13) were significantly greater than those recorded in the preceding months. In *F. fimetaria*, the average clutch size of 13.03 ± 0.68 did not fluctuate significantly across the 6 months.

### Hatching rate

The average hatching rate for clutches laid at week 4 of the first-generation tetracycline treatments was 56.67 ± 3.06%. Hatching rate was not found to be significantly affected by dose in the tetracycline treatments (*F*_4,55 _= 1.82, *P *= 0.14). However, dose was significant for hatching rate in the rifampicin populations (*F*_4,55 _= 2.76, *P *= 0.04). Hatching rates at week 4 of the first generation fell from around 50% for doses of 0.03% and 0.3% to just 28.2 ± 3.9% for the 2.7% rifampicin dose. Hatching rates were not systematically recorded in the second generation but this rate was observed to decline rapidly to zero in the 2.7% rifampicin treatments.

### Nymph:adult ratio

Marked demographic changes occurred because of prolonged exposure to high doses of rifampicin. In the first generation, the average nymph:adult ratio (in which higher values indicate a younger, faster-growing population) of the tetracycline treatments was very similar to that of rifampicin treatments (9.12 ± 0.31 versus 8.95 ± 0.29). Four weeks into the second generation, the average nymph:adult ratio in the tetracycline treatments was 12.31 ± 0.48, reflecting the increase expected from young, newly founded populations. In contrast, the nymph:adult ratio in the second generation of rifampicin populations at the same stage had dropped to just 6.96 ± 0.67. This treatment difference in week 4 of the second generation was already highly significant (*F*_1,118 _= 42.13, *P *< 0.001) and it had become absolute by 8 weeks, when nymphs were entirely absent from all the populations exposed to 2.7% rifampcin and 11 of the 16 populations exposed to 0.9% rifampicin. The individuals of these populations were both devoid of *Wolbachia *and totally sterile.

During the 6-month observation period, the nymph:adult ratio remained at 0 for the entire period in *F. candida *(that is, zero viable nymphs emerged from the hundreds of eggs that were laid). In *F. fimetaria*, nymphs of all instars were present for all 6 months: the nymph:adult ratio did not differ significantly across months, being 3.53 ± 0.10 on average.

### Males

Although males were easily identified in populations of *F. fimetaria *(the sexual species), they were never found in any *F. candida *population.

## Discussion

The elimination of microbial symbionts is likely to be responsible for the sterility observed in *F. candida*. (Although we showed that sustained heavy doses of antibiotic certainly have negative physiological effects, the fact that total sterility persists long after the antibiotic treatment has ended demonstrates that the sterility is unlikely to be due to antibiotic toxicity in itself.) Host sterility is thus attributable to the removal of a microbial strain that facilitates parthenogenesis and/or other reproductive processes. We were careful to distinguish between the facilitation of parthenogenetic diploidisation of the zygote and other non-parthenogenetic reproductive development because it is known that *Wolbachia *can affect either of these discrete processes. For example, in several *Trichogramma *wasp species, *Wolbachia *is responsible for gamete duplication (the parthenogenetic developmental step that leads to a viable zygote) [6], while, in *Asobara tabida*, it has the even more fundamental role of facilitating successful oogenesis by inhibiting cell death [20,21]. We can rule out the possibility that *Wolbachia *plays a role in oogenesis in *F. candida *because of the continued production of sterile eggs in the face of long-term exposure to rifampicin.

In the current system, we cannot unequivocally attribute the identity of the sterility-associated microbe to *Wolbachia*: such a definitive identification could only be made if we had demonstrated that infection of an uninfected collembolan population with *Wolbachia *would lead to parthenogenesis and/or restoration of reproductive ability. Nevertheless, there exists excellent evidence to indicate that *Wolbachia *is the likely culprit. Firstly, we can rule out another common bacterial agent of reproductive alternation, *Cardinium*, because this symbiont is absent in *F. candida *[22]. In *F. candida*, *Wolbachia *bacteria are extremely abundant within both the early oocyte and the embryo, and cytological observations hint that *Wolbachia *could possibly be involved in producing a diploid zygote through the prevention of the separation of sister chromatids at meiosis II [17]. We thus suggest that the sterility we observed occurs because *Wolbachia *plays the crucial role of inducing parthenogenesis. An alternative explanation is that *F. candida *is independently able to produce a diploid zygote but that *Wolbachia *is an obligate mutualist with a crucial role in reproduction, regardless of whether this reproduction is asexual or sexual.

Rifampicin had a negative influence on clutch size in *F. candida*. We interpret this effect to be a consequence of physiological impairments that were not unexpected: rifampicin is known to affect mitochondrial RNA synthesis in eukaryotes at very high concentrations [23]. However, we note that, while clutch sizes increased to normal levels within 1 month of cessation of the rifampicin treatment, total sterility continued. This observation indicates that, while the toxic effects of rifampicin impaired reproductive physiology, these effects are not the cause of sterility. While a similar negative effect on clutch size was not mirrored in *F. fimetaria*, our casual observations lead us to suggest that the high dose of rifampicin was also physiologically detrimental in this species but that other traits, which were not systematically measured, tended to be affected.

The mode of action of tetracycline is to inhibit protein synthesis by preventing the association of aminoacyl-tRNA with the bacterial ribosome [24]. The mode of action of rifampicin is to disrupt an even earlier step in the process of protein synthesis: rifampin binds to prokaryotic DNA-dependent RNA polymerase, inhibiting transcription of DNA to messenger RNA and thereby precluding subsequent translation of RNA to proteins [25]. This different mode of action may be the cause of rifampicin's efficacy in curing *F. candida *of *Wolbachia*.

Although this study found no evidence for the existence of males of *F. candida*, it remains possible that such males do occur rarely in natural populations [9]. If males are ever found or, indeed, if females of *F. candida *are able to cross with males from a sibling species that is yet to be described [10], F. Frati, personal communication], future experiments may shed valuable light on whether *Wolbachia *infection (or its absence) can influence the possibility of sexual reproduction in the extremely common study species that is *F. candida*.

This newly discovered role for *Wolbachia *as an obligate component of reproduction in a diplodiploid animal broadens the already diverse repertoire of phenomena that has been created through the co-evolution of these bacteria and their multitude of hosts. The obligate symbiosis between *F. candida *and *Wolbachia *is not seen in other diplodiploid hosts such as the termite *Reticulitermes speratus*, which is able to continue to reproduce by an independent method of parthenogenesis even after it has been cured of *Wolbachia *[18].

## Conclusion

Examination of the available evidence on the underlying cause of the observed post-antibiotic sterility suggests that, contrary to prior belief, endosymbiont-facilitated parthenogenesis may not be functionally restricted to haplodiploids. Future work will shed light on the exact mechanism by which microbial endosymbionts can enable parthenogenetic reproduction in diplodiploids. The most likely agent of this reproductive facilitation is *Wolbachia*. The *Wolbachia *associated with *F. candida *belongs to supergroup E and the current results give the first indication of what effect this discrete clade of endosymbionts has on its hosts. Future work may even yield evidence that the widespread and adaptable symbionts that are *Wolbachia *have achieved yet another evolutionary feat: usurpation of sperm's role of inducing an egg to commence embryonic development.

## Methods

### Study species

Four clonal populations of *F. candida *(each established from a single parthenogenetic female) were used in experiments involving the administration of antibiotics. The founding individuals were collected from different geographically isolated field populations in the UK and France. Populations were fed on a paste of baker's yeast and kept in sealed containers on a substrate of 10% charcoal, 90% plaster of Paris at a constant temperature of 21 ± 0.2°C and a relative humidity of 100%.

The sibling species, *Folsomia fimetaria*, which reproduces exclusively by sexual means [17,26] was obtained from stock populations maintained at the National Environmental Research Institute, Silkeborg, Denmark. This species was used as a speculative negative control for PCR analyses to detect the presence of *Wolbachia*. Speculation that this species does not host *Wolbachia *was based on the observations of Frati *et al*. [10] that sexually reproducing collembolans that are highly morphologically similar to *F. candida *are devoid of endosymbiotic *Wolbachia*.

### Antibiotic treatment protocols

Two antibiotics, tetracycline (Sigma-Aldrich) and rifampicin (Sigma-Aldrich), which are known to have differing modes of action, were combined with the yeast paste food source and administered *ad libitum *to experimental populations of *F. candida*. For each antibiotic, five dose treatments (measured in percentage dry weight, antibiotic:yeast) were imposed: 0% (control), 0.03%, 0.3%, 0.9% and 2.7%. Each of these five dose treatments was replicated across four populations within each of the four clones of *F. candida *to give a total number of 160 experimental populations. After 4 weeks of antibiotic exposure, 20 first-instar nymphs were taken from each experimental population to give rise to a second generation of 160 experimental populations. These second-generation nymphs were thus guaranteed to be the progeny of mothers that had been exposed to the antibiotic environment for at least 18 days prior to laying.

For all experimental populations from both generations, exact population counts were made on the same day of every week for the first 4 weeks of treatment. Clutch size within each replicate population was estimated by isolating a single adult in a separate rearing container and counting the number of eggs in the first batch of eggs laid. Hatching rate was calculated by observing the proportion of first instar nymphs that emerged from 120 to 150 eggs randomly selected from each replicate population.

Genital examination was used to assess the presence of males. For each population, 20 randomly selected adults were killed and the terminal portion of the abdomen was mounted in lactophenol such that the fifth ventral tergite was clearly visible under ×450 magnification.

A 6-month long observation comparing reproduction in populations of *F. candida *and *F. fimetaria *under exposure to 2.7% rifampicin was also conducted. For the first 3 months of this 6-month period, five populations (with starting sizes of about 100 individuals) of each species were exposed to rifampicin. (This exposure was a continuation of a rifampicin treatment which had been initiated some 4 months earlier.) For the final 3 months of the 6-month period, the antibiotic was no longer included in their food source. Each month, the clutch size of one adult female from each of the eight populations was counted. The proportion of nymphs to adults was also calculated for each population on a monthly basis.

### PCR assays for presence of *Wolbachia*

For *F. candida*, 10 randomly selected adults from each of the following three second-generation treatments were subjected to a process of total DNA extraction followed by selective amplification: (i) control (that is, no antibiotic exposure); (ii) exposure to 2.7% tetracycline; and (iii) exposure to 2.7% rifampicin. In addition, a fourth group of 10 *F. fimetaria *adults was also analysed. Each individual was squashed with forceps and incubated at room temperature in 50 μl of aqueous buffer (10 mM Tris, 1 mM ethylenediaminetetraacetic acid and 25 mM sodium chloride), which also contained 0.2 mg/ml proteinase K. After 30 minutes incubation time, the proteinase K was inactivated by heating the mixture to 95°C for 2 minutes. Two primers named V1 (5'-TTGTAGCCTGCTATGGTATAACT-3') and V6 (5'-GAATAGGTTATGATTTTCATGT-3'), which are specific for the 16S rDNA of all known strains of *W. pipientis *[13], were used in the PCR amplifications. Amplification reactions of 25 μl final volume were created by adding 2 μl from the extraction to a solution containing 250 μM of each deoxyribonucleotide triphosphate, 12.5 pM of the two primers and 1 U AmpliTaq DNA polymerase (Applied Biosystems). The amplification profile was that used by Frati *et al*. [10], and consisted of an initial denaturation at 95°C for 2 minutes, followed by 35 cycles of 45 seconds of denaturation at 95°C, 45 seconds of annealing at 52°C and 50 seconds of extensions at 72°C, with a final extension period of 7 minutes at 72°C. Electrophoresis of amplification products was conducted on agarose gels containing ethidium bromide.

## Abbreviations

PCR: polymerase chain reaction;

## Authors' contributions

NP conceived the research and its design, contributed to the experiments, conducted the molecular assays, undertook data analysis and wrote the manuscript. RK contributed to the research design, undertook the experiments and contributed to the manuscript and data analysis.
